# Supplementary material for: D3MI: an efficient and powerful federated imputation method for bias reduction in the analysis of distributed incomplete data by accounting for within-site correlation and between-site heterogeneity
Source: medRxiv. 2025 May 8:2025.05.08.25327224. Preprint. [Version 1] doi: 10.1101/2025.05.08.25327224 (PMC12083571; doi:10.1101/2025.05.08.25327224)
Supplement: Supplement 1 [file media-1.docx]

Supplementary Material for “D3MI: an efficient and powerful federated imputation method for bias reduction in the analysis of distributed heterogeneous incomplete data”

# **RELATED WORK**

We provide a brief review of existing methods in multilevel multiple imputation for clustered data and computational algorithms associated with GLMM and distributed GLMM.

### **Multilevel imputation**

Missing data is prevalent in clustered data and appropriate imputation algorithms need to account for the multilevel structure.[1, 2] Under centralized settings, a number of multilevel imputation methods have been proposed and implemented. Existing approaches fall into two categories, namely multilevel imputation by joint modeling and by fully conditional specification.[1] The former works by specifying a single model for all incomplete variables. These imputation algorithms are implemented in the R packages pan and jomo based on Bayesian (generalized) linear mixed models.[3, 4] We refer interested readers to a body of literature on this topic.[1, 3-7] The fully conditional specification approach, on the other hand, iterates univariate multilevel imputation over the variables with missingness. In our case, that is a separate GLMM for each incomplete variable.[8] For each variable with missingness, after a GLMM model is fitted, the next step is to generate random draws of the model parameters. Bayesian Markov chain Monte Carlo (MCMC) method can be used but it can require many iterations of intensive computations for the Markov sequence to converge to generate one draw.[9, 10]. Another option is to leverage the large sample normal approximation of the model parameters,[11] however this method (“2l.norm” in the mice package) only applies to continuous missing variables.[10] An approximate Bayesian approach that requires no iteration for drawing a random set of imputation model parameters has been approached.[9, 10] This method works for both continuous and binary variables with missingness, has been implemented in the mice package in R (method “2l.lmer” and “2l.bin”), and has shown good performance.[8]

### The computation associated with GLMMs

Consider the GLMM model given in Equation (1) in the main text. The GLMM model parameters of interest are $\left( \beta_{0},\beta,\sigma_{u}^{2} \right)$. Further, the random effect $u_{i}$ in ($1$) for each hospital $i$ can be subsequently estimated through best linear unbiased prediction. The GLMM model likelihood function has the following form,

$$L\left( \beta_{0},\beta,\sigma_{u}^{2} \right)=\prod_{i=1}^{L} \int_{u_{i}} \prod_{j=1}^{n_{i}} f_{y}\left( {y_{ij}|\beta}_{0},\beta,\sigma_{u}^{2} \right)f_{u}\left( u_{i} | \sigma_{u}^{2} \right)du_{i}, ( SEQ Equation \backslash* ARABIC 2)$$

where $f_{y}(\cdot)$ is the conditional density of the outcome variable $y$ and $f_{u}(\cdot)$ is the normal density function of the random effect $u$. The likelihood function involves integration over the random effects, which has no closed form unless $f_{y}(\cdot)$ is normal, thus the MLE cannot be obtained using derivative-based optimization algorithms (gradient descent, Newton-Raphson) directly. One option is to numerically approximate the integral using Laplace approximation (LA) or (adaptive) Gaussian-Hermite quadrature (GHQ) and maximize the approximate likelihood.[12-15] The GHQ uses multiple quadrature points to approximate the integral, which lead to accurate approximations at high computational cost. The LA can be viewed as a special case of the GHQ with one quadrature point, which trades off some accuracy for better efficiency. In practice, the R packages lme4, glmmML and glmmTMB use these approximations. These packages adopt different strategies to maximize the approximate likelihood function. lme4[16] primarily uses derivative-free “black-box” optimizers such as the Nelder-Mead algorithm and BOBYQA.[17, 18] The glmmML package[19] and the glmmTMB package[20] use derivative-based optimization with the latter relying on automatic differentiation. Another option is the penalized quasi-likelihood (PQL) approach that uses a first-order Taylor expansion to approximate a GLMM as a linear mixed model (LMM).[21] It is implemented in the R function glmmPQL in the MASS package.[22] The PQL works well when the distribution of the outcome conditional on the random effects is approximately normal.[12] Last but not least, one more option to deal with such integration is to set up an expectation-maximization (EM) algorithm, where the hospital-specific random effects are treated as the latent variable. A Monte Carlo EM algorithm to maximize GLMM likelihood functions has been proposed.[12, 23] and implemented in the MCMCglmm package in R.[24] This algorithm is considered technically challenging and the computation is very expensive [25].

### **Distributed GLMMs**

Motivated by the restrictions that individual patient data cannot always be shared across hospitals, privacy-preserving distributed GLMM algorithms have been developed. These algorithms are based on different non-distributed GLMM algorithms described in the previous paragraph, hence inherit their respective strengths and limitations. In addition, to yield comparable estimates to the original non-distributed algorithms, the number of communications and the amount of data that need to be transmitted can also be significantly different. We here analyze the computation and communication efficiency of these distributed GLMM algorithms since both are crucial to distributed multiple imputation. First, a distributed version of the Bayesian EM algorithm is proposed.[26] It involves the Metropolis-Hastings algorithm in the E-step and the Newton-Raphson algorithm in the M-step. Both require a round of communication for each update iteration, and the convergence can take up to thousands of iterations for the Metropolis-Hastings algorithm. A second study on distributed GLMM uses a distributed PQL approach (dPQL).[27] The authors show a lossless property of the algorithm, that is, within a few iterations, the dPQL estimates for the GLMM can be almost exactly the same as in the (centralized) PQL estimates for the same model. The communication cost is also relatively low, involving a square matrix and a vector that both have the same dimensionality as the data and a scalar in each iteration. The downside is that the estimates can be inaccurate when the conditional distribution of the outcome variable given the random effects is far from normal. Nonetheless, the PQL approach remains an appealing method for its simplicity and efficiency. Last but not least, a distributed algorithm is developed based on the maximum likelihood approach following the LA and adaptive GHQ approximation.[28] This algorithm is based on the algorithm used by the glmmML package. The algorithm involves evaluating high-order derivatives, which can be numerically unstable. The simulation results show that the algorithm requires up to hundreds of iterations to converge, which means hundreds of rounds of communication, and the estimates have considerable amount of bias. Finally, it is worth mentioning that there has not been a distributed GLMM based on the algorithm used in lme4 that is generally viewed as the go-to package for fitting GLMMs, likely due to the technical difficulty associated with building a distributed derivative-free optimizer.

# **REFERENCES**

1 Van Buuren S. Multiple imputation of multilevel data. Handbook of advanced multilevel analysis: Routledge, 2011:173-96.

2 Van Buuren S. *Flexible imputation of missing data*: CRC press, 2018.

3 Quartagno M, Grund S, Carpenter J. Jomo: a flexible package for two-level joint modelling multiple imputation. R Journal 2019;**9**(1).

4 Schafer J, Zhao J. pan: multiple imputation for multivariate panel or clustered data (R package version 1.4), 2016.

5 Yucel RM. Random covariances and mixed-effects models for imputing multivariate multilevel continuous data. Statistical modelling 2011;**11**(4):351-70.

6 Carpenter JR, Bartlett JW, Morris TP, et al. *Multiple imputation and its application*: John Wiley & Sons, 2023.

7 Goldstein H, Carpenter JR, Browne WJ. Fitting multilevel multivariate models with missing data in responses and covariates that may include interactions and non-linear terms. Journal of the Royal Statistical Society Series A: Statistics in Society 2014;**177**(2):553-64.

8 Van Buuren S, Groothuis-Oudshoorn K. mice: Multivariate imputation by chained equations in R. Journal of statistical software 2011;**45**:1-67.

9 Jolani S. Hierarchical imputation of systematically and sporadically missing data: an approximate Bayesian approach using chained equations. Biometrical Journal 2018;**60**(2):333-51.

10 Jolani S, Debray TP, Koffijberg H, et al. Imputation of systematically missing predictors in an individual participant data meta‐analysis: a generalized approach using MICE. Statistics in medicine 2015;**34**(11):1841-63.

11 Resche-Rigon M, White IR. Multiple imputation by chained equations for systematically and sporadically missing multilevel data. Statistical methods in medical research 2018;**27**(6):1634-49.

12 McCulloch CE. *Generalized linear mixed models*: Ims, 2003.

13 Shun Z, McCullagh P. Laplace approximation of high dimensional integrals. Journal of the Royal Statistical Society Series B: Statistical Methodology 1995;**57**(4):749-60.

14 Rabe-Hesketh S, Skrondal A, Pickles A. Reliable estimation of generalized linear mixed models using adaptive quadrature. The Stata Journal 2002;**2**(1):1-21.

15 Pinheiro JC, Chao EC. Efficient Laplacian and adaptive Gaussian quadrature algorithms for multilevel generalized linear mixed models. Journal of Computational and Graphical Statistics 2006;**15**(1):58-81.

16 Bates D, Maechler M, Bolker B, et al. Package ‘lme4’. convergence 2015;**12**(1):2.

17 Nelder JA, Mead R. A simplex method for function minimization. The computer journal 1965;**7**(4):308-13.

18 Powell MJ. The BOBYQA algorithm for bound constrained optimization without derivatives. Cambridge NA Report NA2009/06, University of Cambridge, Cambridge 2009;**26**:26-46.

19 Broström G, Holmberg H. Generalized linear models with clustered data: Fixed and random effects models. Computational Statistics & Data Analysis 2011;**55**(12):3123-34.

20 Brooks ME, Kristensen K, Van Benthem KJ, et al. glmmTMB balances speed and flexibility among packages for zero-inflated generalized linear mixed modeling. The R journal 2017;**9**(2):378-400.

21 Breslow NE, Clayton DG. Approximate inference in generalized linear mixed models. Journal of the American statistical Association 1993;**88**(421):9-25.

22 Ripley B, Venables B, Bates DM, et al. Package ‘MASS’. CRAN R 2013;**538**:113-20.

23 Booth JG, Hobert JP. Maximizing generalized linear mixed model likelihoods with an automated Monte Carlo EM algorithm. Journal of the Royal Statistical Society Series B: Statistical Methodology 1999;**61**(1):265-85.

24 Hadfield JD. MCMC methods for multi-response generalized linear mixed models: the MCMCglmm R package. Journal of statistical software 2010;**33**:1-22.

25 Bolker BM, Brooks ME, Clark CJ, et al. Generalized linear mixed models: a practical guide for ecology and evolution. Trends in ecology & evolution 2009;**24**(3):127-35.

26 Zhu R, Jiang C, Wang X, et al. Privacy-preserving construction of generalized linear mixed model for biomedical computation. Bioinformatics 2020;**36**(Supplement_1):i128-i35.

27 Luo C, Islam MN, Sheils NE, et al. dPQL: a lossless distributed algorithm for generalized linear mixed model with application to privacy-preserving hospital profiling. Journal of the American Medical Informatics Association 2022;**29**(8):1366-71.

28 Li W, Tong J, Anjum MM, et al. Federated learning algorithms for generalized mixed-effects model (GLMM) on horizontally partitioned data from distributed sources. BMC Medical Informatics and Decision Making 2022;**22**(1):269.
